# Dioecious *Silene latifolia *plants show sexual dimorphism in the vegetative stage

**DOI:** 10.1186/1471-2229-10-208

**Published:** 2010-09-20

**Authors:** Jitka Zluvova, Jiri Zak, Bohuslav Janousek, Boris Vyskot

**Affiliations:** 1 Department of Plant Developmental Genetics, Institute of Biophysics AS CR v. v. i., Kralovopolska 135, 612 65 Brno, Czech Republic

## Abstract

**Background:**

Prior to this study, no differences in gene expression between male and female dioecious plants in the vegetative state had been detected. Among dioecious plants displaying sexual dimorphism, *Silene latifolia *is one of the most studied species. Although many sexually dimorphic traits have been described in *S. latifolia*, all of them are quantitative, and they usually become apparent only after the initiation of flowering.

**Results:**

We present RT-PCR-based evidence that in *S. latifolia*, sexual dimorphism in gene expression is present long before the initiation of flowering. We describe three ESTs that show sex-specific (two male specific and one female specific) transcription at the rosette stage before the first flowering season.

**Conclusions:**

To our knowledge, this study provides the first molecular evidence of early pre-flowering sexual dimorphism in angiosperms.

## Background

Sexual dimorphism (the systematic difference in form or other trait(s) not present in sexual organs between individuals of different sex in the same species) is a widely studied phenomenon in animal models [1] and in humans [2]. Much less is known about sexual dimorphism in vascular dioecious plants (reviewed in [3]).

Among vascular plants displaying sexual dimorphism, *Silene latifolia *is (together with *Fragaria virginiana *- [4]) the most studied species. The first study on sexually dimorphic traits in *S. latifolia *was performed already in the 19th century [5], and since that time many sexually dimorphic traits have been described (e. g., [6-9]). However, the only known genes involved in sexual dimorphism are those involved in the control of flower development. During flower development, sexual dimorphism starts to occur very early. At the morphological level, the central zone of the floral meristem is significantly smaller in males than in females [10]. This is caused by cell division arrest in male tissues [11]. The difference between male and female flower bud morphology is preceded by differences at the gene expression level. Developmental pathways involved in the switch of male or female flower program have been also identified [12,13].

The differential expression of some genes probably results from different modes of selection operating in males and females: males are limited in their reproductive success by access to mates, whereas females are more limited by resources [14]. In animals, the evolution of the sexual dimorphism is primarily driven by competition between males and selection for traits recognized by females as marks of male fitness (reviewed in [15])]. Similar principles are probably also at work in animal pollinated plants. In *S. latifolia*, odor-compounds involved in pollinator attraction differ significantly between sexes, suggesting that selection for higher attractiveness among competing males is mediated by the sensory ecology of the pollinator [16]. In addition, males produce on average up to 16 times more flowers than pollinated females [17]. This difference in flower number is probably driven by a combination of male competition, and, at least partly, by a higher consumption of resources by developing seeds in pollinated female flowers, which probably results in a trade off between seed size and flower number. The difference in flower number is, indeed, less pronounced in non-pollinated females, which produce on average 4 times fewer flowers than males [17,18]. Yet, selection for increased flower number in males is hypothesized to be the primary mechanism for the further evolution of sexual dimorphism in other traits [8]. It also seems reasonable to expect that differences in the vegetative parts of plants evolved in concert with different flowers types or architecture of inflorescences carried by the plant [3]. Dawson and Geber [3] pointed out that many sexually dimorphic traits could evolve as a consequence of their correlation with other sexually dimorphic traits and so they need not be of adaptive value. Correlations between flower size and the size of the stem leaves have been reported by several independent studies (reviewed by [3]). Steven *et al. *[18] suggested that variation in sex-limited genes with pleiotropic effects and/or linkage between sex limited loci occurs in *S. latifolia*. They statistically predicted that selection for increased flower numbers in males along with weak selection for increased flower size in females could lead to dimorphic evolution in several other traits including leaf mass [18].

Almost all of the sexually dimorphic traits in *S. latifolia *described so far become apparent only after the initiation of flowering. Notable exceptions to this pattern include: sex-dimorphism in the long-term survival of buried seeds and burial induced dormancy in *S. latifolia *[19], sex-dimorphism in emergence time [20] and in the time to flowering [20,21]. We present the first molecular evidence that sexually dimorphic gene expression is present in *S. latifolia *even at the rosette stage, a long time before the initiation of flowering, and describe three ESTs with sex-specific gene expression.

## Results and discussion

**Figure 1:**
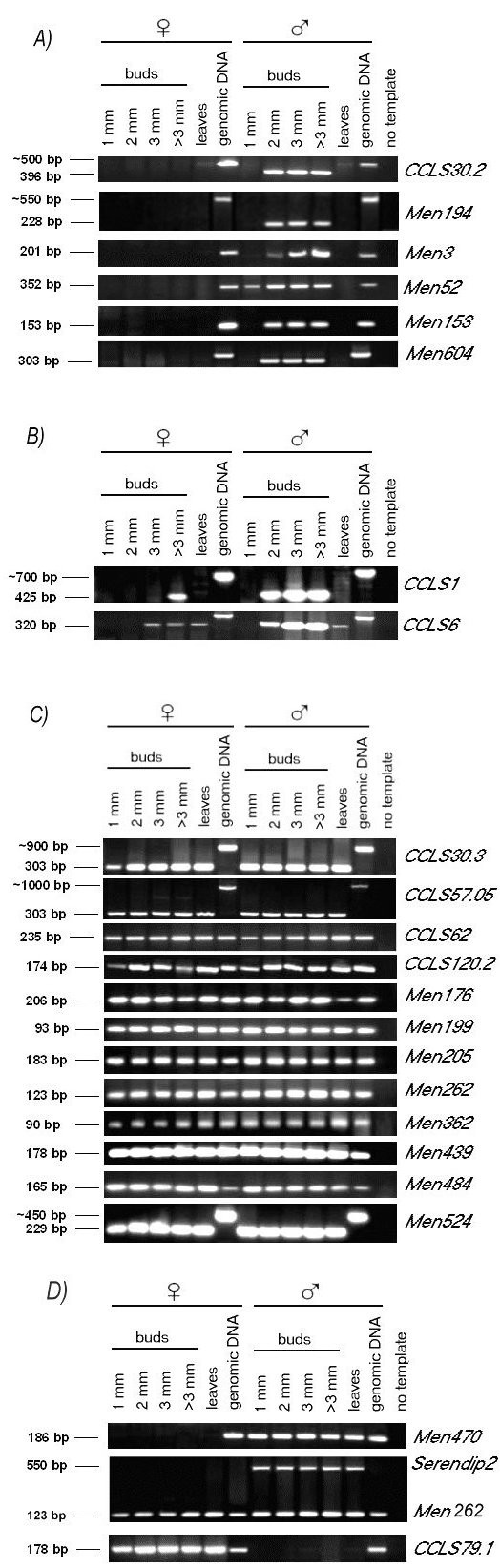
**RT-PCR analyses of all the studied ESTs**. Expression was investigated by RT-PCR analysis on the tissues indicated above each lane. The ESTs amplified are indicated on the right. Male buds of two mm length represent the stage when meiosis starts in anthers. Female meiosis starts in eight mm long female buds. Further details concerning the flower development in *S. latifolia *are summarized in Additional file 6: Supplementary table S4 (according to Farbos *et al. *[47]). (A) ESTs expressed exclusively in male flower buds. (B) ESTs starting to be expressed in male flower buds earlier than in female flower buds. (C) ESTs expressed in leaves and flower buds of both sexes. (D) ESTs showing sex specific expression in all the studied tissues. Two of them (*Serendip2*, and *Men-470*) are showing male specific expression while *CCLS79.1 *gene shows female specific expression. *Men-262 *is included to illustrate that *Serendip2 *is amplified with the same pair of primers and it serves also as a proof of the sufficient quality of templates.

We re-tested the expression patterns of 22 available *S. latifolia *ESTs previously described according to Northern blots or Virtual Northerns [22-24] as preferentially expressed in male flowers and/or early stamen (for the list of the ESTs chosen for this study, see Additional file 1: Supplementary table S1). Fewer genes than previously claimed have expression limited to male flower buds suggesting the importance of RT-PCR analyses in this case. Only six out of 15 genes previously described as male flower bud specific were expressed in male flower buds only and not in the leaves or in female flower buds (Figure 1A). Two genes, originally described as male flower bud specific, were expressed in male flower buds earlier than in female flower buds (Figure 1B). Twelve genes were expressed in all samples tested (Figure 1C). For the comparison of the previously published data and our results see Additional file 2: Supplementary table S2. We also found one EST *(Men-470) *expressed exclusively in male flower buds and leaves and one EST *(CCLS79.1) *expressed exclusively in female flower buds and leaves (Figure 1D). We also serendipitously found one new 550 bp long EST as a "by-product" of PCR amplification of *Men-262 *(Figure 1D) and named it *Serendip2 *[GenBank: GU120088]. *Serendip2 *was expressed exclusively in male flower buds and leaves.

To elucidate the discrepancy between the expression patterns found here and those presented in previous research [22-24], we performed a homology search of the studied ESTs followed by a search for putative orthologous sequences in *Arabidopsis thaliana *(for the results, see Additional file 1: Supplementary table S1 and Additional file 3: Supplementary figures S1-11) and their expression patterns according to Genevestigator V3 [25,26] (for the results, see Additional file 1: Supplementary table S1; Genevestigator workspace file is included as Additional file 4). We found that the gene expression patterns described in *A. thaliana *were not in a contradiction to our results (cf. Additional file 1: Supplementary table S1 and the Figure 1).

In this article, we present sex-specific expression patterns of *Men-470, Serendip2 *and *CCLS79.1*; they are expressed in a sex-specific manner not only in flower buds, but also in the leaves of plants in the vegetative stage of development. At this stage, no expression of genes involved in the flower formation is present in *A. thaliana *(reviewed in [27]). *Men-470 *has already been studied using RT-PCR by other authors [24], who reported expression both in male and female flowers and in leaves (sex not specified). Using our primers, we observed transcription in males in all tissues studied, but no transcription in females. This result suggests that the expression of the copy amplified by our primer pair is already sex specific in the rosette stage. We can exclude the alternative possibility that our primers amplified only the Y-linked copy of *Men-470 *by amplifying the sequence from females (XX). *Serendip2 *is a new EST that was found in this study. *Serendip2 *has an open reading frame along the whole sequence, but it has no homology to any known gene either at the DNA or protein level. The most interesting case is probably the *CCLS79.1 *gene. This sequence was amplified from genomic DNA samples of both females and males, suggesting that the difference in expression is not caused by sex linkage. The results we obtained in *CCLS79.1 *differ from the results obtained previously in Northern blots [22] that showed expression both in male and female flower buds and no expression in male leaves. The different results of our study can be explained by the fact that RT-PCR can selectively amplify one of several copies present in the genome.

Our results clearly show that male and female *S. latifolia *plants differ in the expression of at least three genes long before the initiation of flowering; this situation is analogous to the pregonadal stage in mammals [28]. Growth differences and sex specific expression are present in mammalian embryos in the preimplantation stage, long before the formation of sex organs. Similarly, sex-dimorphic gene expression has been found in gastrulating chicken embryos [29]. The main difference between plant and animal bodies is that plants do not possess a true germline and sexual organs develop relatively late in plant life. Given this pattern, our discovery of sexual dimorphism at the rosette stage is surprising. Indeed, contrary to animals, sexual dimorphism in plants at the vegetative stage before inflorescence initiation seems to be extremely rare. In classical dioecious model species, the earliest differences between male and female individuals are apparent in the inflorescence shape (in hop *(Humulus) *or hemp (*Cannabis*)). Spatial sex segregation, which is caused by differential seed germination and seedling survival (reviewed by [30]), is an indirect indication of the existence of sexual dimorphism in the vegetative stage of plants [31]. Additionally, the salt grass *Distichlis spicata*, a species characterized by spatial segregation of sexes, shows sex specific differences in susceptibility to colonization by a mycorrhizal fungus [30]. In this species, the sex specific differences even result in a strong inter-sexual competition [32]. As already listed in introduction, there are only a few indirect indications of the sexually dimorphic sex expression in the early vegetative state in *S. latifolia *that were obtained by previous studies [19-21]. The differences in expression patterns of three ESTs found in this study are the first qualitative differences between the sexes in the vegetative stage in *S. latifolia*. They are also the first described sequences in plants connected with the sexual dimorphism in the vegetative stage.

The existence of the sex specifically expressed genes in *S. latifolia *in the rosette stage suggests that there may be some, as yet undetected, physiological differences between sexes. We speculate that such hidden sexual dimorphism may be present in many dioecious species, and this study should inspire other scientists to test other dioecious species for sexual dimorphism in early vegetative stages. The existence of sexually dimorphic patterns means that the *S. latifolia *plants "know" their sex a long time before flowering, and this situation probably enables the plants to prepare for flowering in a sex specific manner.

## Conclusions

To our knowledge, this study provides the first molecular evidence of early pre-flowering sexual dimorphism in angiosperms.

## Methods

### Orthology identification and search for the expression patterns in *A. thaliana*

Orthology data were obtained from OrthoMCL [33]. Files containing protein sequence data and orthology group information were downloaded from OrthoMCL version 4, currently containing genes from nine green plant genomes. The OrthoMCL clustering method provides a convenient, but necessarily imperfect means of estimating orthology and paralogy ([34], [35]). The highest BLAST hits can, in some cases, occur due to domain homologies, rather than homology to orthologs [36]. We performed phylogenetic analysis to avoid this misleading orthologue identification. Sequences for phylogenetic tree construction were obtained via a BLASTX homology search of the database of non-redundant protein sequences at NCBI (nr). All ESTs showing significant homology to known sequences were subjected to phylogenetic tree construction to further confirm their orthologues in other plant species. For the phylogenetic analysis, translated sequences were aligned using ClustalW version 1.83 [37], and the alignment was manually corrected using Seaview [38]. Ambiguously aligned parts of the sequences were excluded using Gblocks [39]. Phylogenetic trees were constructed by the maximum likelihood algorithm using PhyML version 3.0.1 [40] using the LG + Γ4 + I model [41] and they were visualized using Dendroscope [42]. Branches were tested for reliability by approximate likelihood-ratio test [43]. Phylogenetic trees were rooted using an appropriate outgroup. Putative orthologues in *A. thaliana *were detected at all *A. thaliana *genes that grouped with a respective *Silene latifolia *EST with a high degree of confidence. The cut-off to detect orthology was set to 0.7. The expression patterns of the *A. thaliana *orthologues were searched using Genevestigator V3 [25,26].

### Extraction of nucleic acids and RT-PCR

*S. latifolia *plants were grown as described by Markova et al. [44]. Genomic DNA was isolated as described previously [45]. The sex of the plants was estimated at the rosette stage based on the length polymorphism between X and Y copy of the gene SlssX/Y (using the primers c2B12+1 and c2B12-2)[46]. RNA from a bulk sample of six male or female plants was isolated from rosette leaves (before the first flowering season; at the eight leaves stage) and flower buds of four different sizes (smaller than 1 mm, between 1 and 2 mm, between 2 and 3 mm and bigger than three mm). For RNA isolation and reverse transcription, we used the same procedures as described previously [12]. To verify the results, RT-PCR was also performed on a single male and a single female with the same results as the bulk analysis. A list of all PCR primers and conditions used in this study is provided in Additional file 5: Supplementary Table S3. PCR products were analyzed on agarose gels and visualized under UV light in the presence of ethidium bromide. Expression was classified in a qualitative manner as present or absent.

## Authors' contributions

JZl conceived and designed the experiments. JZl, JZa and BJ performed the experiments. JZl analyzed the data. JZl, BJ and BV contributed reagents/materials/analysis tools. BV discussed the paper. JZl and BJ wrote the paper. All authors read and approved the final manuscript.

## Supplementary Material

Additional file 1 **Table S1**. Complete list of the studied genes with available information concerning putative *A. thaliana *orthologues found in this study. Click here for file

Additional file 2 **Table S2**: Comparison of the previously published results with the results obtained in this study The file compares the gene expression data obtained in this study with the previously published data. The published data on testing of Y-chromosome linkage are also summarized. Click here for file

Additional file 3 **Figures S1-11**. The file contains these supplementary figures: Supplementary figure S1 - Phylogenetic analysis of the gene CCLS6. Supplementary figure S2 - Phylogenetic analysis of the gene CCLS30.2. Supplementary figure S3 - Phylogenetic analysis of the gene CCLS30.3. Supplementary figure S4 - Phylogenetic analysis of the gene CCLS57.05. Supplementary figure S5 - Phylogenetic analysis of the gene CCLS62. Supplementary figure S6 - Phylogenetic analysis of the gene CCLS120.2. Supplementary figure S7 - Phylogenetic analysis of the gene Men-194. Supplementary figure S8 - Phylogenetic analysis of the gene Men-439. Supplementary figure S9 - Phylogenetic analysis of the gene Men-484. Supplementary figure S10 - Phylogenetic analysis of the gene Men-524. Supplementary figure S11 - Phylogenetic analysis of the gene Men-604 Click here for file

Additional file 4 **Genevestigator workspace file for all the found putative *A. thalina *orthologs**. This file contains expression data available for the found putative *A. thaliana *orthologs of the genes: *CCLS6*, *CCLS30.2*, *CCLS30.3*, *CCLS57.05*, *CCLS62*, *CCLS120.2*, *Men-194*, *Men-439*, *Men-484*, *Men-524 *and *Men-604*. The file can be viewed using Genevestigator V3 at the URL: https://www.genevestigator.com/gv/user/gvLogin.jsp (Registration is recommended.) Click here for file

Additional file 5 **Table S3**. List of PCR primers and conditions Click here for file

Additional file 6 **Supplementary Table S4: Flower development in *Silene latifolia***. The table compares development of male and female flowers in *S. latifolia*. Click here for file
