# Supplementary material for: Dioecious Silene latifolia plants show sexual dimorphism in the vegetative stage
Source: BMC Plant Biol. 2010 Sep 20;10:208. doi: 10.1186/1471-2229-10-208 (PMC2956557; doi:10.1186/1471-2229-10-208)
Supplement: Additional file 1 — Table S1. Complete list of the studied genes with available information concerning putative A. thaliana orthologues found in this study. [file 1471-2229-10-208-S1.DOC]

**Supplementary table S1 -Complete list of the studied genes with available information concerning putative *A. thaliana* orthologues found in this study**

| *S. latifolia* EST [Ref.] | orthologs in *A. thaliana* | Description | expression in *A. thaliana* |
| --- | --- | --- | --- |
| *CCLS1* [1] | no homology | N/A | N/A |
| *CCLS6* [1] | *At1g02050* | chalcone and stilbene synthase family protein | preferentially inflorescence and flower |
|  | *At4g00040* | chalcone and stilbene synthase family protein | ubiquitous |
| ***CCLS30.2* [1]** | ***At1g62940*** | **4-coumarate-CoA ligase family protein** | **inflorescence, flower and stem** |
| ***CCLS30.3* [1]** | ***At2g05630*** | **autophagy-associated Atg8 family protein** | **ubiquitous** |
| ***CCLS57.05* [1]** | ***At3g55960*** | **NLI interacting factor (NIF) family protein** | **ubiquitous** |
| *CCLS62* [1] | *At5g64640* | pectinesterase family protein | ubiquitous, preferentially stamen and pollen |
|  | *At5g09760* | pectinesterase family protein | ubiquitous |
| *CCLS79.1* [1] | no homology | N/A | N/A |
| ***CCLS120.2* [1]** | ***At5g04550*** | **unknown function** | **ubiquitous** |
| *Men-3* [2] | no homology | N/A | N/A |
| *Men-52*[3] | no homology | N/A | N/A |
| *Men-153* [3] | no homology | N/A | N/A |
| *Men-176* [3] | no homology | N/A | N/A |
| ***Men-194* [3]** | ***At1g62940*** | **4-coumarate-CoA ligase family protein** | **inflorescence, flower and stem** |
| *Men-199* [3] | no homology | N/A | N/A |
| *Men-205* [3] | no homology | N/A | N/A |
| *Men-262* [3] | no homology | N/A | N/A |
| *Men-362* [3] | no homology | N/A | N/A |
| *Men-439* [3] | *At3g15450* | unknown function | ubiquitous |
|  | *At4g27450* | unknown function | ubiquitous |
| *Men-470* [3] | no homology | N/A | N/A |
| *Men-484* [3] | *At1g79550* | cytosolic phosphoglycerate kinase (PGK) | ubiquitous with the exception of pollen |
|  | *At1g56190* | phosphoglycerate kinase, putative | ubiquitous |
|  | *At3g12780* | nuclear phosphoglycerate kinase | ubiquitous |
| ***Men-524* [3]** | ***At2g30200*** | **[acyl-carrier-protein] S-malonyltransferase** | **ubiquitous with the exception of pollen** |
| ***Men-604* [3]** | ***At1g33430*** | **galactosyltransferase family protein** | **inflorescence, flower, stamen, stem** |
| *Serendip2* | no homology | N/A | N/A |

Notes: The ESTs for which the orthologue was unambiguously found, are in bold.

No homologous sequences were identified in *S. latifolia* with the exception of *CCLS1* (1), which shows homology to *Men-7*(2).

**References:**

1. Barbacar N, Hinnisdaels S, Farbos I, Moneger F, Lardon A, Delichere C, Mouras A, Negrutiu I: **Isolation of early genes expressed in reproductive organs of the dioecious white campion *(Silene latifolia)* by subtraction cloning using an asexual mutant.** *Plant J* 1997, **12:** 805-817.

2. Scutt CP, Li T, Robertson SE, Willis ME, Gilmartin PM **Sex determination in dioecious *Silene latifolia*. Effects of the Y chromosome and the parasitic smut fungus *(Ustilago violacea)* on gene expression during flower development.** *Plant Physiol* 1997, **114:**969-979.

3. Scutt CP, Jenkins T, Furuya M, Gilmartin PM: **Male specific genes from dioecious white campion identified by fluorescent differential display.** *Plant Cell Physiol* 2002, **43:**563-572.
