# Supplementary material for: Dioecious Silene latifolia plants show sexual dimorphism in the vegetative stage
Source: BMC Plant Biol. 2010 Sep 20;10:208. doi: 10.1186/1471-2229-10-208 (PMC2956557; doi:10.1186/1471-2229-10-208)
Supplement: Additional file 2 — Table S2: Comparison of the previously published results with the results obtained in this study The file compares the gene expression data obtained in this study with the previously published data. The published data on testing of Y-chromosome linkage are also summarized. [file 1471-2229-10-208-S2.DOC]

**Supplementary table S2 -Comparison of previously published results with the results obtained in this study**

| **Gene [Ref.]** | **Y-linked copy?** | **Sex specific expression?** | |
| --- | --- | --- | --- |
|  |  | **previously published data** | **this study** |
| *CCLS1* [1] | no [1] | Yes | Yes (time difference) |
| *CCLS6* [1] | no [1] | Yes | Yes (time difference) |
| *CCLS30.2* [1] | no [1] | Yes | Yes |
| *CCLS30.3* [1] | no [1] | Yes | No |
| *CCLS57.05* [1] | no [1] | No | No |
| *CCLS62* [1] | no [1] | No | No |
| *CCLS79.1* [1] | no [1,4] | No | Yes |
| *CCLS120.2* [1] | no [1] | Yes | No |
| *Men-3* [2] | no [1] | Yes | Yes |
| *Men-52* [3] | no [1] | Yes | Yes |
| *Men-153* [3] | multiple copies; one of them Y specific [3] | Yes | Yes |
| *Men-176* [3] | no [3] | Yes | No |
| *Men194* [3] | no [3] | Yes | Yes |
| *Men-199* [3] | no [3] | Yes | No |
| *Men-205* [3] | no [3] | Yes | No |
| *Men-262* [3] | no [3] | Yes | No |
| *Men-362* [3] | no [3] | Yes | No |
| *Men-439* [3] | no [3] | No | No |
| *Men-470* [3] | multiple copies; one of them Y specific [3] | Yes (semi-quantitative difference) | Yes |
| *Men-484* [3] | no [3] | No | No |
| *Men-524* [3] | no [3] | No | No |
| *Men-604* [3] | no [3] | Yes | Yes |
| *Serendip2* | not analysed | N/A | Yes |

**References:**

1. Barbacar N, Hinnisdaels S, Farbos I, Moneger F, Lardon A, Delichere C, Mouras A, Negrutiu I: **Isolation of early genes expressed in reproductive organs of the dioecious white campion *(Silene latifolia)* by subtraction cloning using an asexual mutant.** *Plant J* 1997, **12:** 805-817.

2. Scutt CP, Li T, Robertson SE, Willis ME, Gilmartin PM **Sex determination in dioecious *Silene latifolia*. Effects of the Y chromosome and the parasitic smut f fungus *(Ustilago violacea)* on gene expression during flower development.** *Plant Physiol* 1997, **114:**969-979.

3. Scutt CP, Jenkins T, Furuya M, Gilmartin PM: **Male specific genes from dioecious white campion identified by fluorescent differential display.** *Plant Cell Physiol* 2002, **43:**563-572.

4. Laporte V, Charlesworth D: **Non-sex-linked, nuclear cleaved amplified polymorphic sequences in Silene latifolia**. *J Hered* 2001, **92**:357-359.
