# Supplementary material for: Dioecious Silene latifolia plants show sexual dimorphism in the vegetative stage
Source: BMC Plant Biol. 2010 Sep 20;10:208. doi: 10.1186/1471-2229-10-208 (PMC2956557; doi:10.1186/1471-2229-10-208)

Supplementary figure S1 - Phylogenetic analysis of the gene *CCLS6*

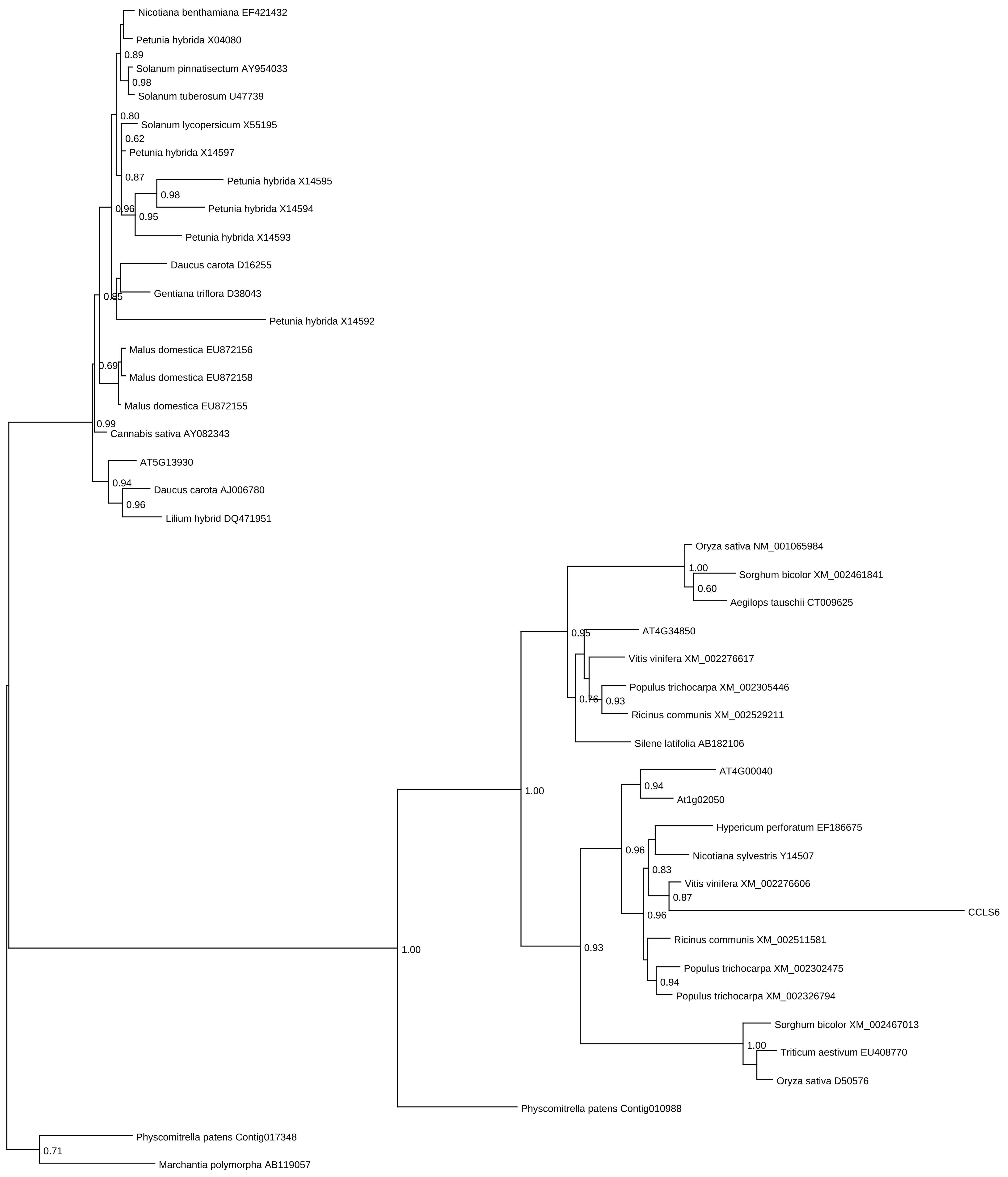

Supplementary figure S2 - Phylogenetic analysis of the gene *CCLS30.2*

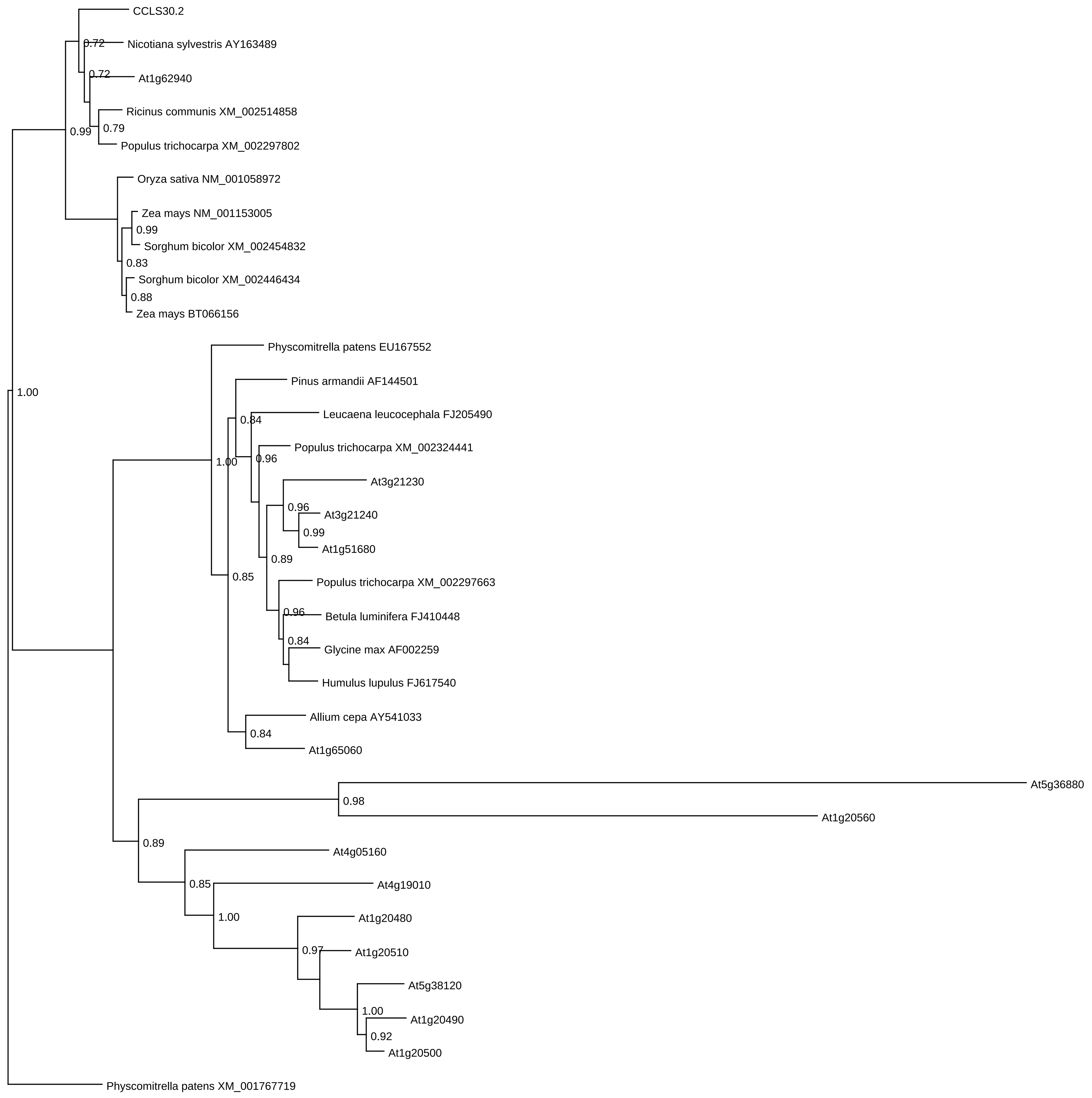

Supplementary figure S3 - Phylogenetic analysis of the gene *CCLS30.3*

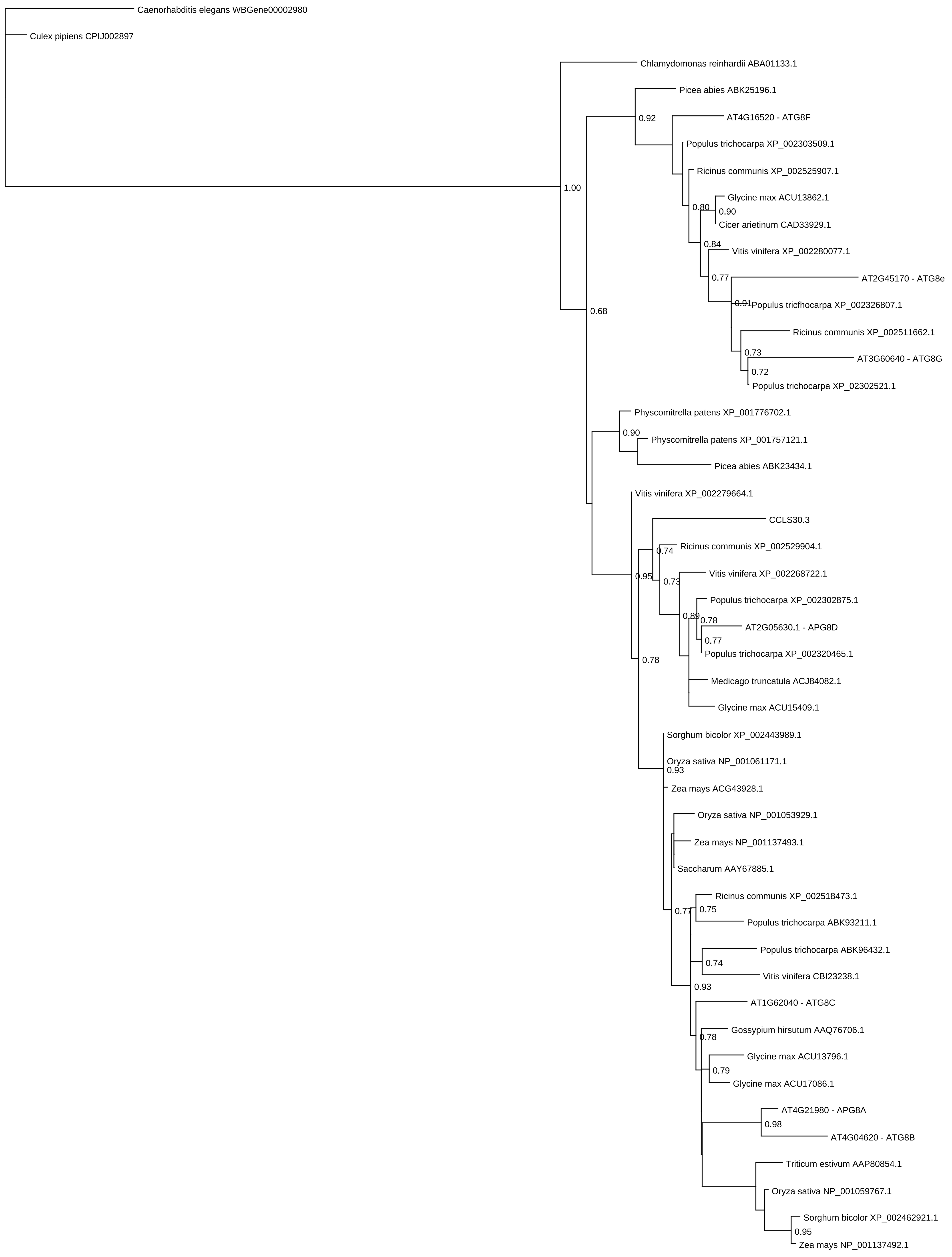

Supplementary figure S4 - Phylogenetic analysis of the gene *CCLS57.05*

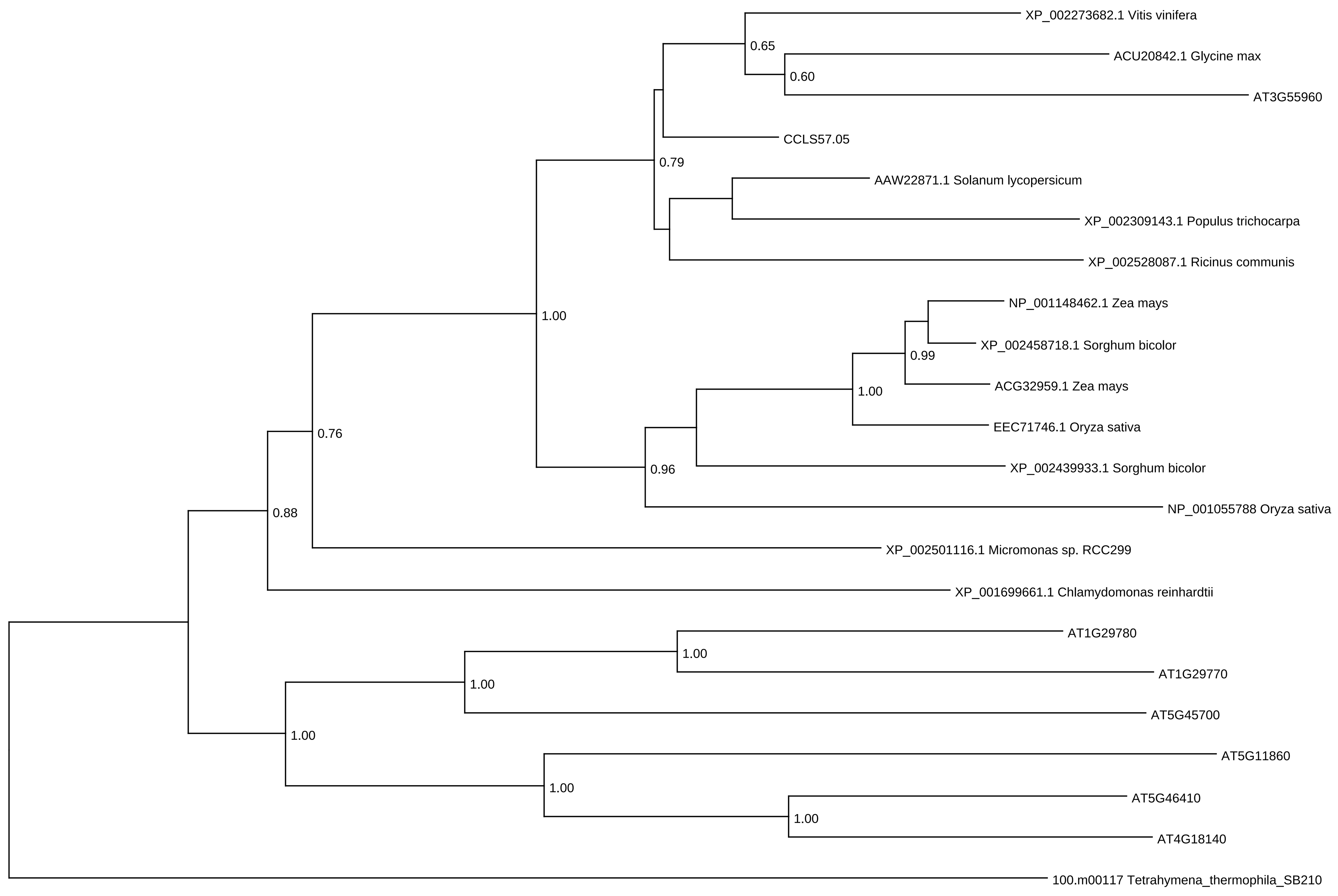

Supplementary figure S5 - Phylogenetic analysis of the gene *CCLS62*

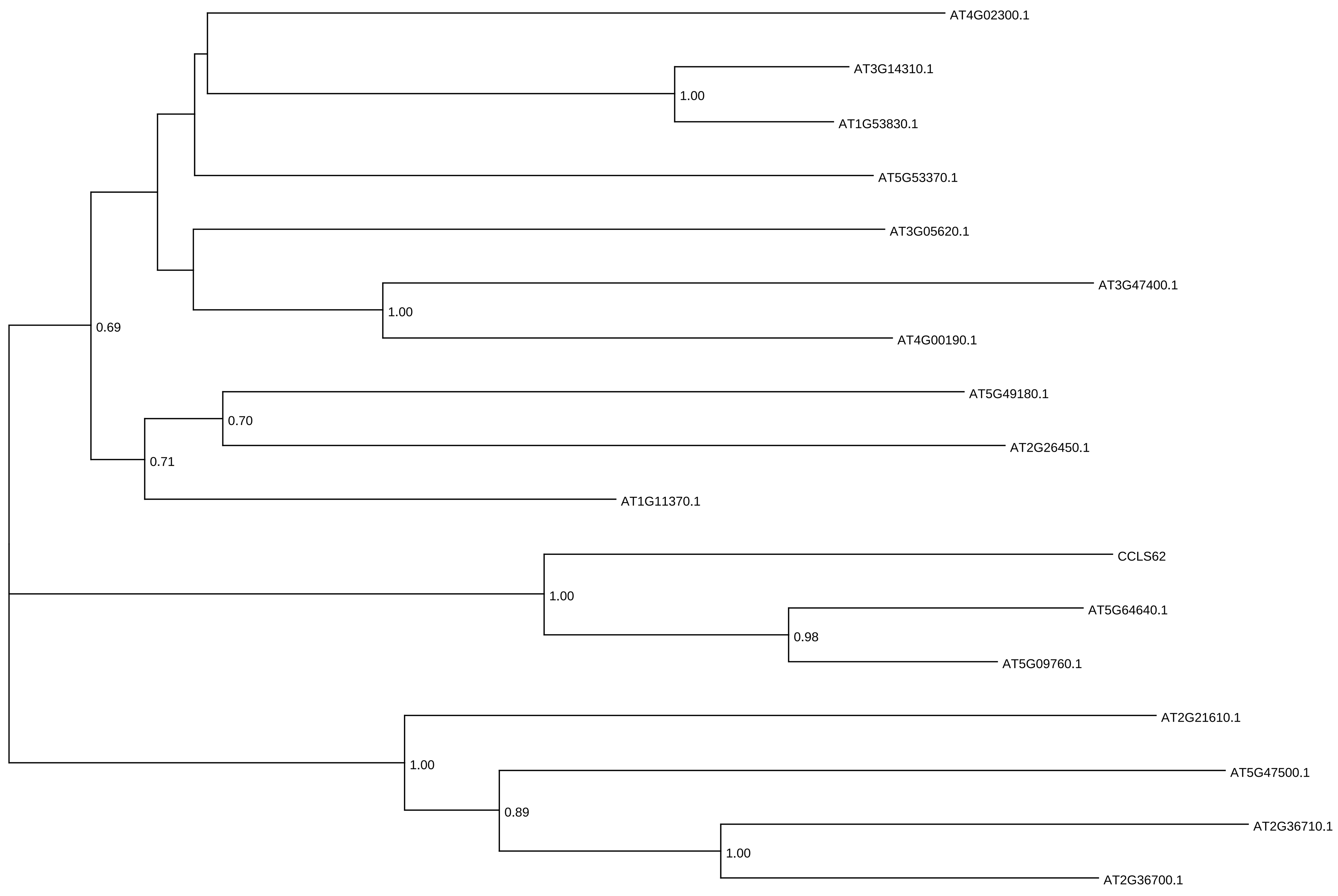

Supplementary figure S6 - Phylogenetic analysis of the gene *CCLS120.2*

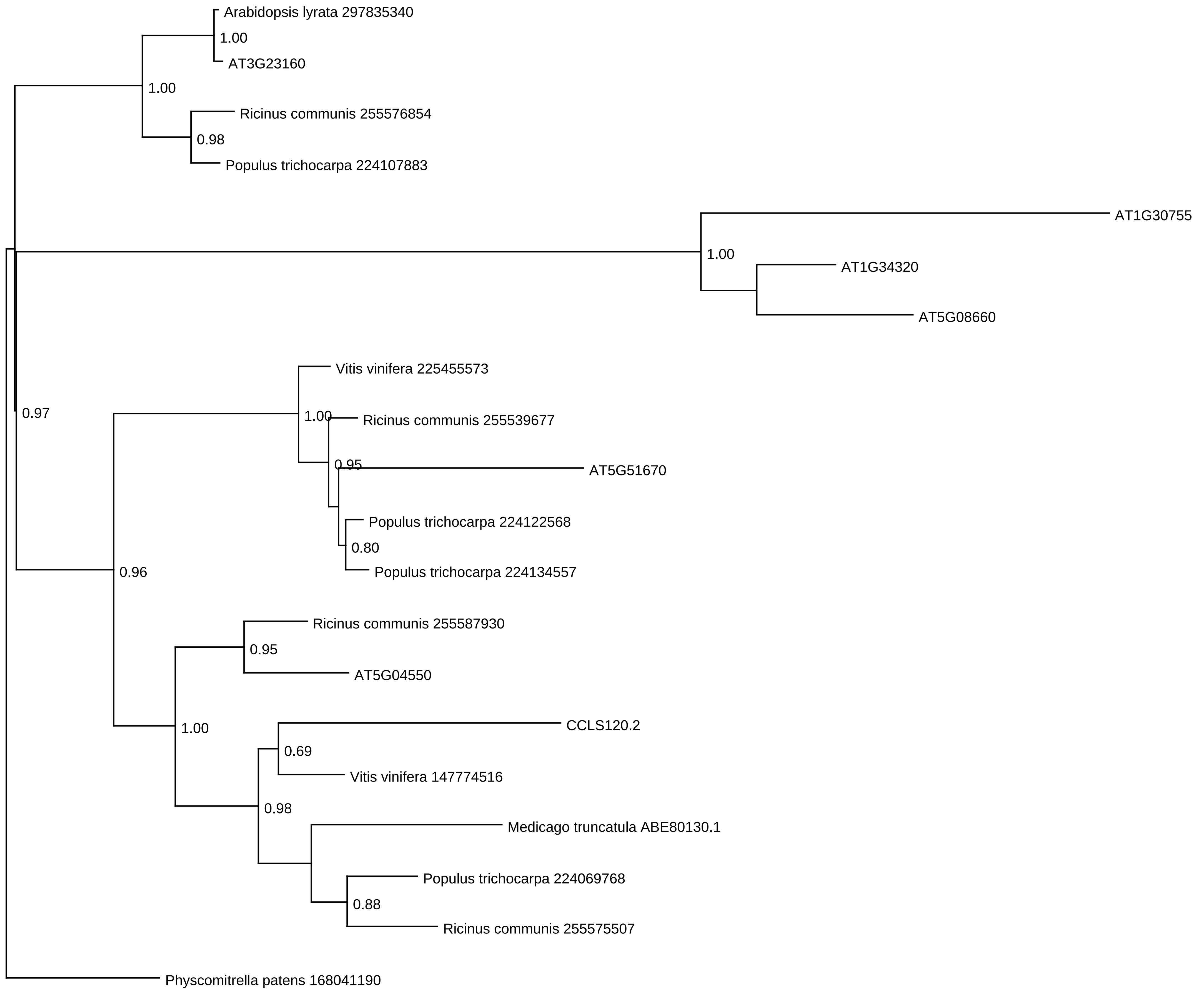

Supplementary figure S7 - Phylogenetic analysis of the gene *Men-194*

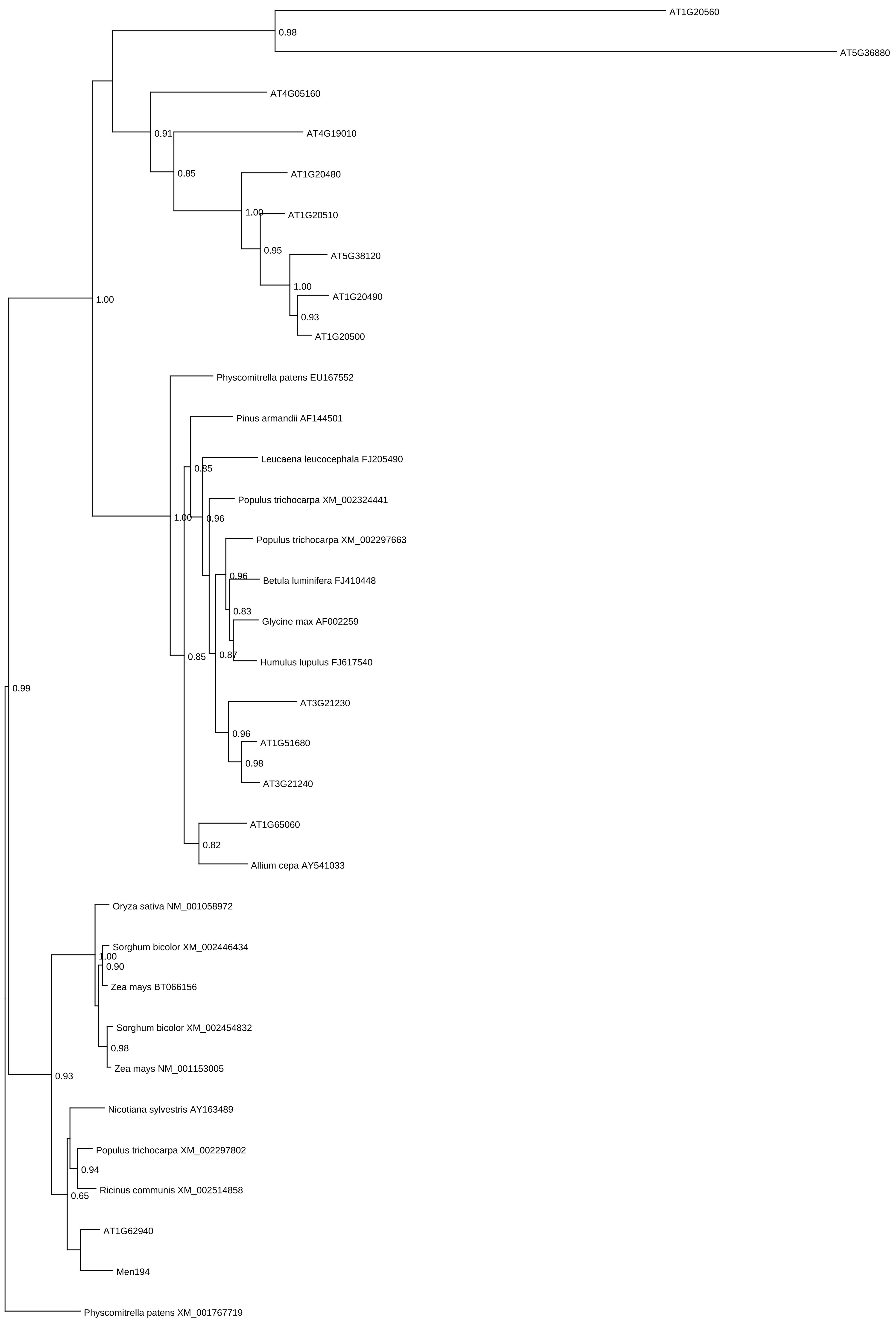

Supplementary figure S8 - Phylogenetic analysis of the gene *Men-439*

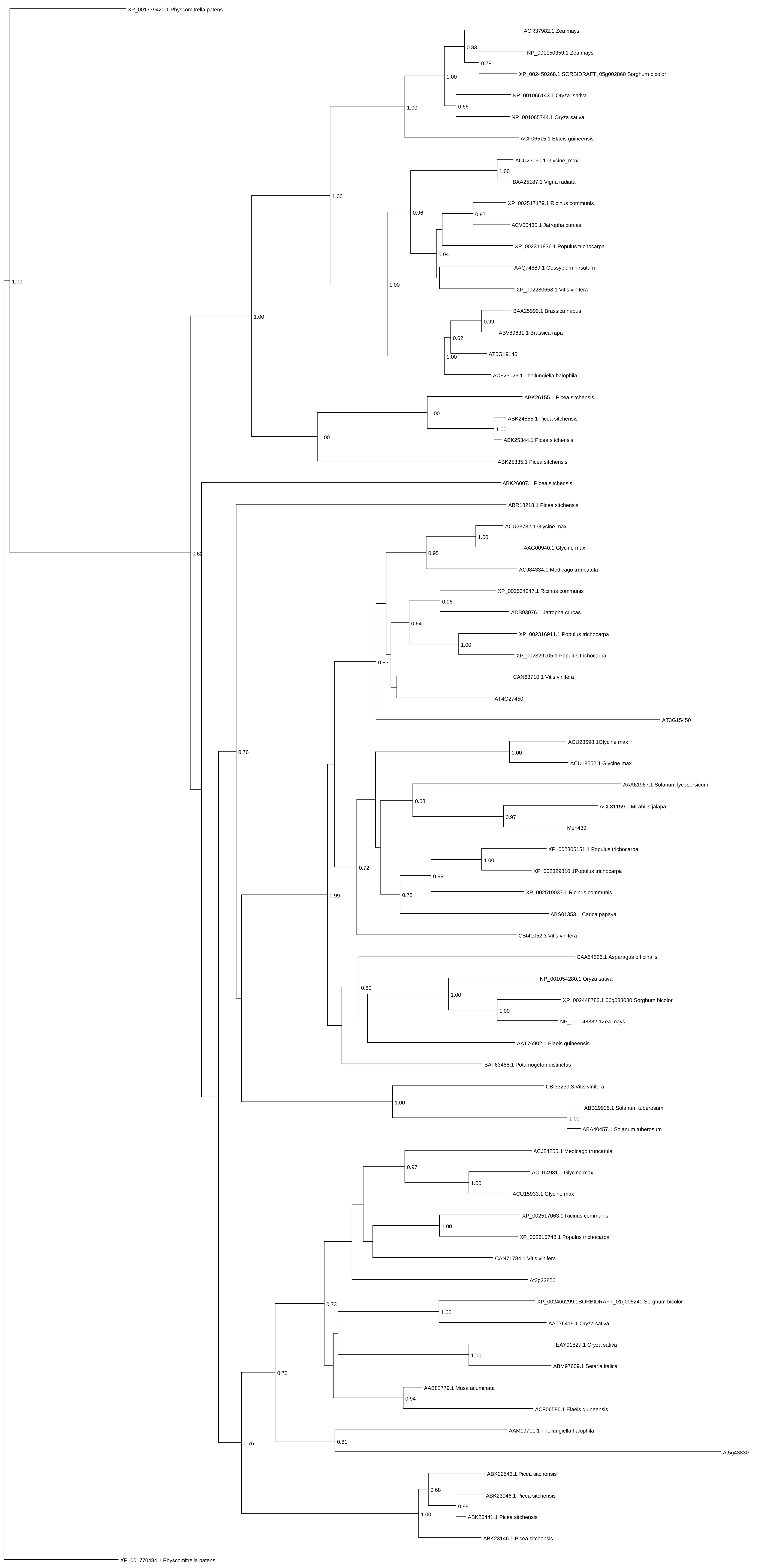

Supplementary figure S9 - Phylogenetic analysis of the gene *Men-484*

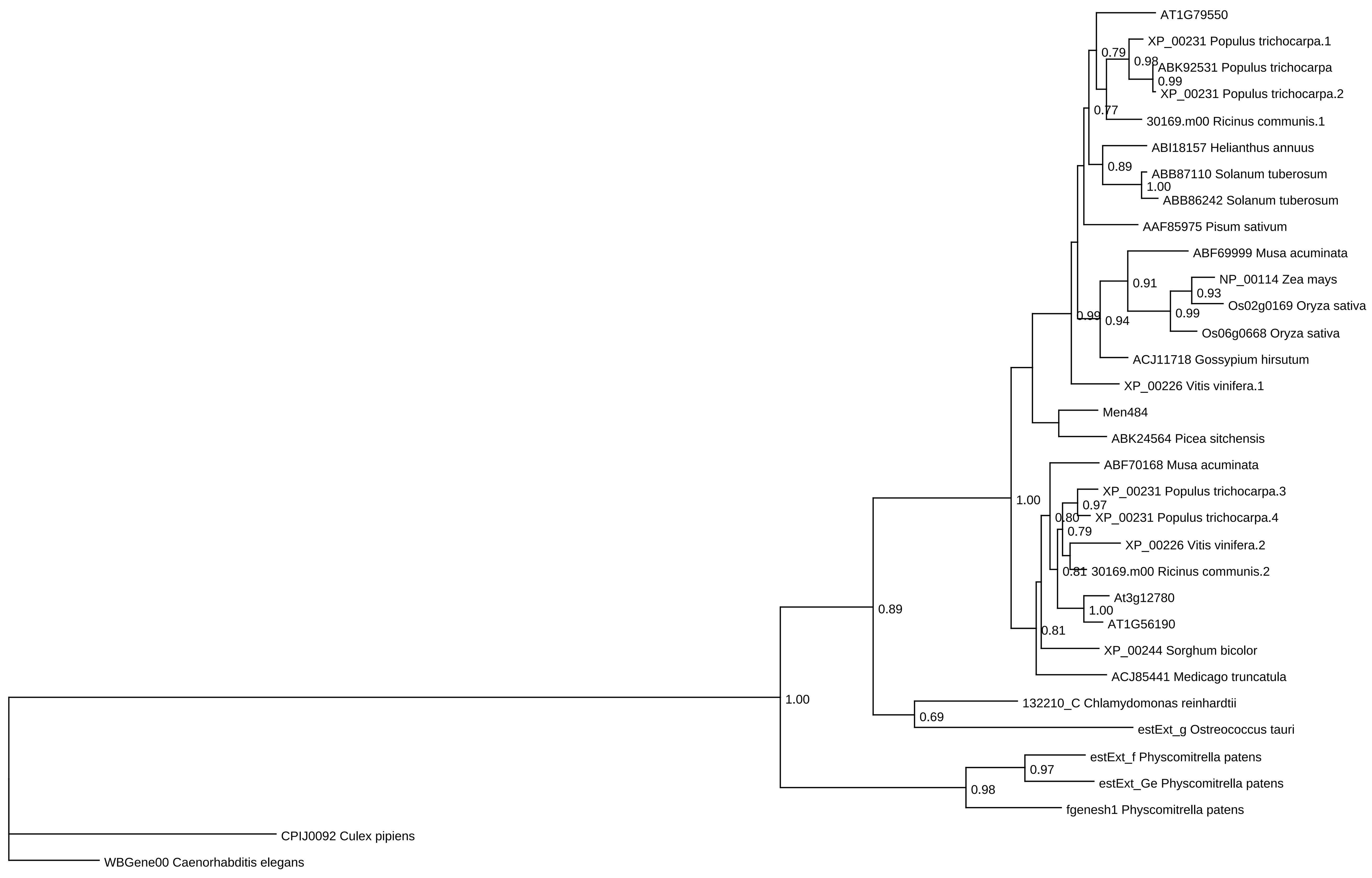

Supplementary figure S10 - Phylogenetic analysis of the gene *Men-524*

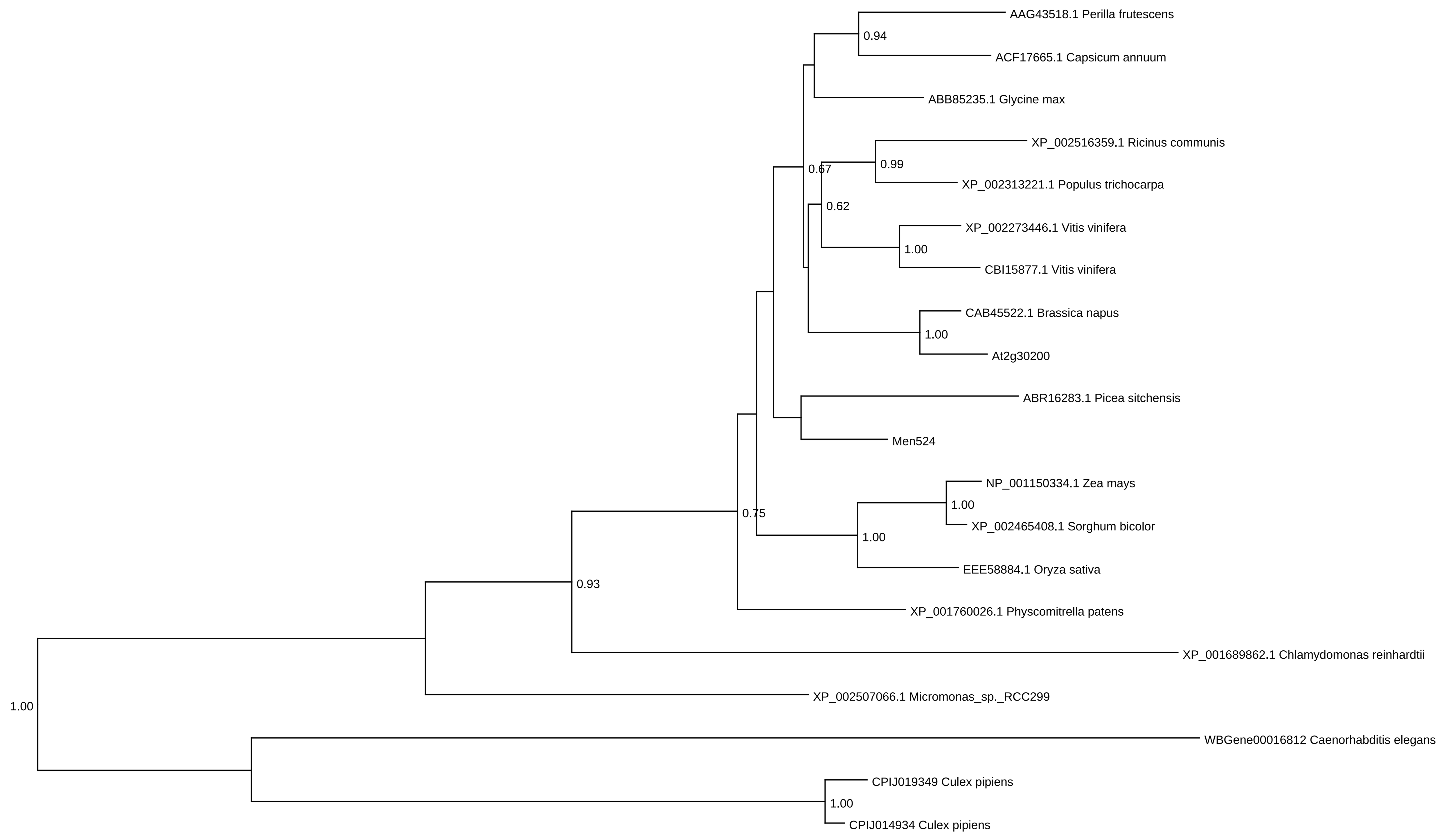

Supplementary figure S11 - Phylogenetic analysis of the gene *Men-604*

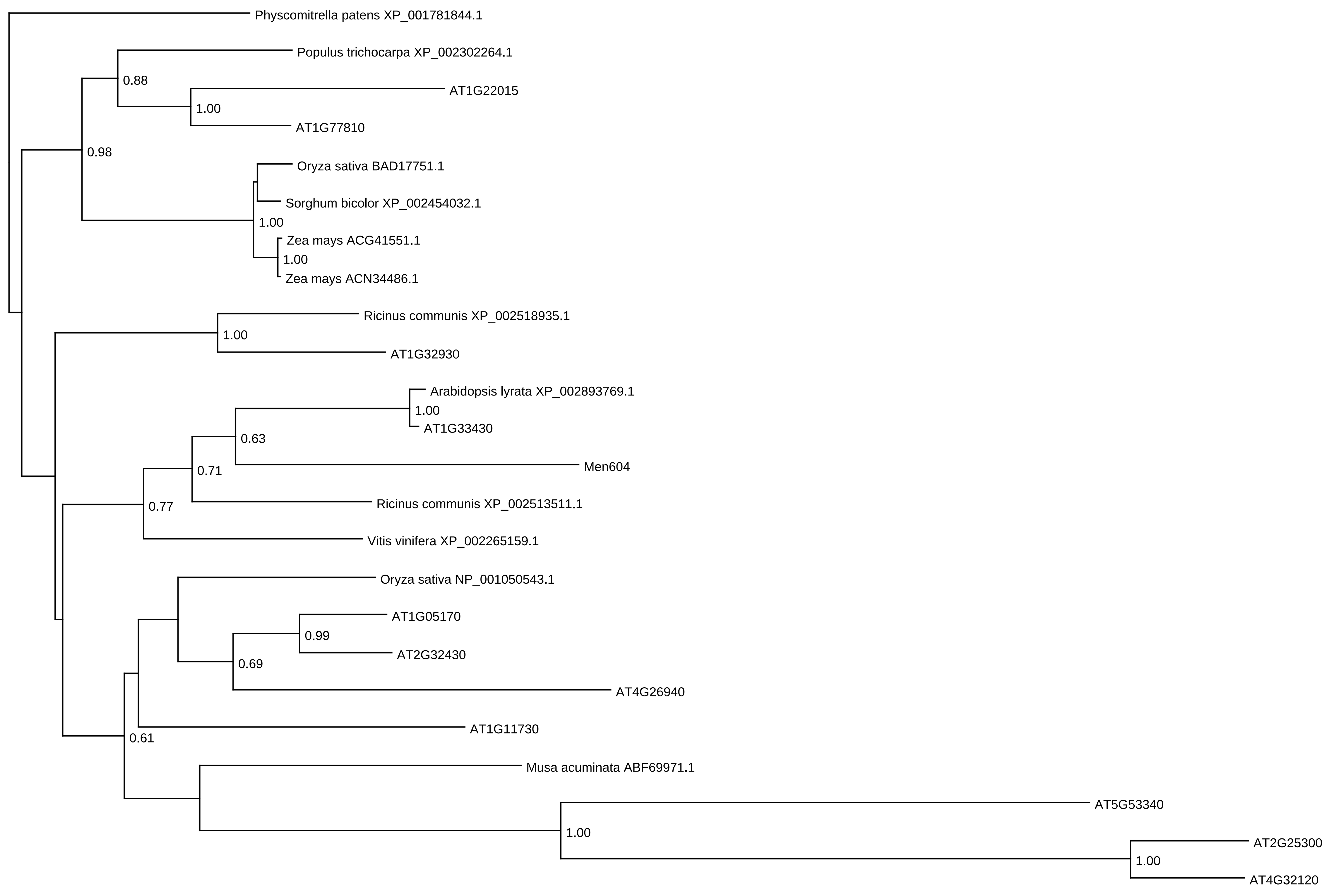

Supplement: Additional file 3 — Figures S1-11. The file contains these supplementary figures: Supplementary figure S1 - Phylogenetic analysis of the gene CCLS6. Supplementary figure S2 - Phylogenetic analysis of the gene CCLS30.2. Supplementary figure S3 - Phylogenetic analysis of the gene CCLS30.3. Supplementary figure S4 - Phylogenetic analysis of the gene CCLS57.05. Supplementary figure S5 - Phylogenetic analysis of the gene CCLS62. Supplementary figure S6 - Phylogenetic analysis of the gene CCLS120.2. Supplementary figure S7 - Phylogenetic analysis of the gene Men-194. Supplementary figure S8 - Phylogenetic analysis of the gene Men-439. Supplementary figure S9 - Phylogenetic analysis of the gene Men-484. Supplementary figure S10 - Phylogenetic analysis of the gene Men-524. Supplementary figure S11 - Phylogenetic analysis of the gene Men-604 [file 1471-2229-10-208-S3.PDF]
