# Supplementary material for: Dioecious Silene latifolia plants show sexual dimorphism in the vegetative stage
Source: BMC Plant Biol. 2010 Sep 20;10:208. doi: 10.1186/1471-2229-10-208 (PMC2956557; doi:10.1186/1471-2229-10-208)
Supplement: Additional file 5 — Table S3. List of PCR primers and conditions [file 1471-2229-10-208-S5.DOC]

**Supplementary Table S3 -List of PCR primers and conditions.**

A general PCR cycle used was: initial denaturation 94 ^o^C / 3 min, followed by 35 cycles of denaturation 94 ^o^C / 30 sec, annealing T_a_ ^o^C / 1 min, and extension 72 ^o^C / t_e_ sec.

* Touchdown PCR cycle was: initial denaturation 94 ^o^C / 3 min, followed by 10 cycles of denaturation 94 ^o^C / 30 sec, annealing 60 ^o^C / 1 min with the decrement 0.5 ^o^C / cycle, and extension 72 ^o^C / t_e_ sec, followed by 25 cycles of denaturation 94 ^o^C / 30 sec, annealing 55 ^o^C / 1 min, and extension 72 ^o^C / 60 sec.

| EST | Primer | Sequence | T_a_ (^o^C) | t_e_ (s) |
| --- | --- | --- | --- | --- |
| *Men-3* | Men3-F1 | aggcagtggaaatggtcaaggt | 60 | 120 |
|  | Men3-R1 | gtgctctactcttactctgatgc |  |  |
| *Men-52* | Men52-F1 | agtgattccttgacgcaactag | 60 | 60 |
|  | Men52-R1 | ttgtctagccaacacccaaaca |  |  |
| *Men-153* | Men153-F1 | ttattcctaaatcgaatggttga | 60 | 60 |
|  | Men153-R1 | tccattaagaatccgggtga |  |  |
| *Men-176* | Men176-F1 | ctaccaaggtgtttcgtcaacg | 60 | 60 |
|  | Men176-R2 | acggaaactcaaacgataccct |  |  |
| *Men-194* | Men194-F1 | ccctccctaagaacactcctgg | 60 | 60 |
|  | Men194-R1 | tggcctccaattcagctggagc |  |  |
| *Men-199* | Men199-F2 | gacgacatcattgaactagcgt | 60 | 60 |
|  | Men199-R1 | cttagctaacctatgagcggct |  |  |
| *Men-205* | Men205-F1 | tacggcagtaaagccagtgacc | 60 | 60 |
|  | Men205-R2 | tcatgtagcactaaattgtgt |  |  |
| *Men-262* | Men262-F1 | ggccttcaactgcgtcaagg | 60 | 60 |
|  | Men262-R1 | tgacttgagttcaaacctcg |  |  |
| *Men-362* | Men362-F1 | agtaattttgcacctgagctgg | 60 | 60 |
|  | Men362-R1 | agctgtactgaaggggttggag |  |  |
| *Men-439* | Men439-F1 | ggactgacgagtttcgaacacc | 60 | 120 |
|  | Men439-R1 | cttgagatcatgcttgaggacc |  |  |
| *Men-470* | Men470-F1 | aaacgaagcccacttcttca | 60 | 60 |
|  | Men470-R1 | cacggagtgcgttaactgag |  |  |
| *Men-484* | Men484-F1 | tcttgcggataagatgagccac | 60 | 120 |
|  | Men484-R1 | tgttctaatggttacgggcctc |  |  |
| *Men-524* | Men524-F1 | caccattaagaagatactggctcg | 60 | 60 |
|  | Men524-R1 | aagggtaagctagcctctagga |  |  |
| *Men-604* | Men604-F1 | aagttgtgaagacgcatcaagc | 60 | 60 |
|  | Men604-R1 | cacctcctggtgtcgcgctgtg |  |  |
| *CCLS1* | CCLS1-F1 | tggcctcgataaaccaatggca | 60 | 60 |
|  | CCLS1-R1 | gcatattgctgaatgctgatctcc |  |  |
| *CCLS6* | CCLS6-F1 | acaagtggtcgccttccgacac | 65 | 60 |
|  | CCLS6-R1 | tcttcagtaaggcacccatcga |  |  |
| *CCLS30.2* | CCLS30.2-F1 | gcacttgtggaagcgtgttctg | 60 | 60 |
|  | CCLS30.2-R1 | ccacattcgctttaatcggctg |  |  |
| *CCLS30.3* | CCLS30.3-F1 | gcagaagctgctcggctcaagg | 60 | 60 |
|  | CCLS30.3-R1 | tccaaacacgttctctccactg |  |  |
| *CCLS57.05* | CCLS57.05-F1 | cggagagctggttcagtcgtcg | 60 | 60 |
|  | CCLS57.05-R1 | tttggaaccattcgggcatgtg |  |  |
| *CCLS62* | CCLS62-F2 | tgccacccacaattgatttcgt | touchdown* | 60 |
|  | CCLS62-R2 | ccgttttacccataccatccc |  |  |
| *CCLS79.1* | CCLS79.1-F2 | aggaaccatgaagatgctgttgata | 60 | 60 |
|  | CCLS79.1-R2 | taactagccgggtatcaaccat |  |  |
| *CCLS120.2* | CCLS120.2-F2 | acttgcggaattgcttgagcga | 60 | 60 |
|  | CCLS120.2-R2 | ttcccagcctgctcacacactg |  |  |
| *Serendip2* | Men262-F1 | ggccttcaactgcgtcaagg | 60 | 60 |
|  | Men262-R1 | tgacttgagttcaaacctcg |  |  |
