# Supplementary material for: Dioecious Silene latifolia plants show sexual dimorphism in the vegetative stage
Source: BMC Plant Biol. 2010 Sep 20;10:208. doi: 10.1186/1471-2229-10-208 (PMC2956557; doi:10.1186/1471-2229-10-208)
Supplement: Additional file 6 — Supplementary Table S4: Flower development in Silene latifolia. The table compares development of male and female flowers in S. latifolia. [file 1471-2229-10-208-S6.DOC]

**Supplementary Table S4 –Flower development in *Silene latifolia***

| **Length mm** | **female flower** | | **male flower** | |
| --- | --- | --- | --- | --- |
|  | **stage** | **status** | **stage** | **status** |
| not determined | 1 | Vegetative/floral transition | 1 | Vegetative/floral transition |
| not determined | 2 | Inflorescense meristem | 2 | Inflorescense meristem |
| 0.1 | 3 | Floral meristem | 3 | Floral meristem |
| 0.17 | 4 | Sepal primordia initiation | 4 | Sepal primordia initiation |
| 0.25 | 5 | Floritypic stage= sepal primordia clearly visible,  Petal and stamen primordia start to develop | 5 | Floritypic stage= sepal primordia clearly visible,  Petal and stamen primordia start to develop |
| 0.30 | 6.1 | Gynoecium is "mushroom" shaped with five grooved centers (cavities)  Stamen primordia with meristematic tissue, frequent mitoses  Sepals non-overlapping  Petal primordia clearly separated from internal stamen primordia | 6 | External stamen: primordia with meristematic tissue, freguent mitoses  Gynoecium dome initiates to elongate.  Sepals non-overlapping  Petal primordia clearly separated from internal stamen primordia |
| 0.40 |  | Sepals fused = formation of calyx  Petals present as small mounds, alternating with sepals | 7 | External stamen: bilobal stage, filament initialisation, prevascularisation, tapetum initiation  Gynoecium elongation continues  Sepals fused = formation of calyx  Petals present as small mounds, alternating with sepals |
| 0.6 | 6.2 | Gynoecium is "pentagone"-shaped with five distinct locules.  Stamen growth and differentiation slows down |  |  |
| 0.75 |  | Calix closed, beginning of vascularisation  Petals tongue like shaped. | 8 | Stamen filament elongation and vessel formaton, external anther size= 0.2 mm; tetralobal stage, locules formed; internal anthers size = 0.1 mm.  Degenerated gynoecium present as finger like projection, further differentiation is stopped.  Calix closed, beginning of vascularisation  Petals tongue like shaped. |
| 1.0 | 7.1 | Gynoecium in a "barrel and star" stage; barrel shaped ovary with clear septa and placental tissue.  Stamen: stalked filaments; anthers bilobal, primary sporogenous cells present, no tapetum; prevascularisation of the filaments  Sepals dark green; trichome formation | 9.1 | Stamen fill up the calyx space;  external anther size = 0.3 mm;  internal anther size=0.2 mm; pollen mother cells with no callose  Sepals dark green, trichome formation |
| 1.8 |  |  | 9.2 | External anther size = 0.5 mm;  pollen mother cells with callose;  internal anther size=0.3 mm. |
| 2 | 7.2 | Gynoecium wall vascularised;  Ovary: subepidermic meristematic domes at right angle to the placenta  Sepals: elongation  Petals: pale green, ovoid, vascularised | 9.3 | External anthers: size 0.8 mm; meiosis initiated.  Internal anthers: size=0.5 mm pollen mother cells with callose;  Sepals: elongation  Petals: pale green, ovoid, vascularised |
| 2.4 |  |  | 9.4 | External anthers: size 1 mm; telophase II  Internal anthers: size 0.7 mm; meta-anaphase I |
| 2.8 |  | Sepals increase in size  Petals cordiform, pale green; 1 mm long | 10 | External anthers size 1.1 mm; tetrads  Internal anthers size 0.8 mm; anaphase II  Sepals increase in size  Petals cordiform, pale green; 1 mm long |
| 3.0 | 8.1 | Gynoecium elongation; two nrows of ovules per carpel; apical fusion and emergence of styles  Anthers: regression of sporogenous cell diferentiation into parencymatous cells. | 11.1 | External anthers: free microspores; tapetum degeneration  Internal anthers: tetrads |
| 5.0 | 8.2 | Gynoecium: style differentiation  Ovary: archesporial cell; formation of internal and external integuments  Sepal growth accelerated  Petal grows accelerated | 11.2 | External anthers: pollen mitosis  Pollen increase in size  Internal anthers: size 1.1 mm  Sepal growth accelerated  Petal grows accelerated |
| 6.5 | 9 | Gynoecium elongation and vascularisation of the styles  Ovary: elongation of the funiculus  Elongation of petals | 11.3 | Stamen increase in size  External anthers: size 2.1 mm; binucleate pollen  Internal anthers: 1.9 mm  Elongation of petals |
| 8 | 10.1 | Gynoecium differentiation of the stigmatic papillae  Ovary: meiosis; beginning of funiculus vascularisation |  |  |
| 9 | 10.2 | Ovary: Embryosac formation  internal and external integuments of equal length  formation of micropyle  Calyx diameter larger in females.  Petals overgrow the the stamens; | 11.4 | Proportional elongation of the external and internal stamens; connective lysis between locules; lignification of anther walls  External anthers: size 2.2 mm  Internal anthers: 2.1 mm  Pollen maximum size  Calyx diameter smaller in males |
| 11 | 10.3 | Ovary: campylotropous type  Petals: further elongation | 11.5 | External anthers: asynchronous mitoses in the generative nucleus  Sepals: indentation visible in male flowers  Petals: further elongation |
| 13 | 11.1 | Gynoecium: styles and petals reach the same hight  Calyx opening  Corola just visible | 11.6 | Anthers reach maximum size.  Maturation of trinucleate pollen  Calyx opening  Corola just visible |
| 15 |  | Sepals: maximum size  Petals rolled up | 11.7 | Stamen filament elongation  Gynoecium: maximum size of the "finger like projection reached  Sepals: maximum size  Petals rolled up |
| 18 | 11.2 | Gynoecium lignification of outer cell layers at the carpel/style junction;  vascularisation of placenta  Corolla emerging from the calyx | 11.8 | Pollen: carbohydrate storage  Corolla emerging from the calyx |
| 21 | 11.3 | Style elongate rapidly  Petals: maximum size | 11.9 | Stamen: maximum size  Pollen: round; final pollen sculpturing  Petals: maximum size |
| 23 | 11.4 | Ovary increases in size  Petals: unfolding | 11.10 | Dehiscence of external anthers  pollen release in the flower cavity;  Petals: unfolding |
| 25 | 11.5 | Style emerging through the petals  swelling of the ovary | 11.11 | Dehiscence of internal anthers  pollen release in the flower cavity; anthesis |
| 30 | 12 | Gynoecium: maximum size; mature ovules | 12 | Anthers are degenerating |

Notes: Gynoecium development is described in red.

Stamen development is described in blue.

Table is based on the data of Farbos *et al.* [1]

**References:**

1. Farbos I, Oliveira M, Negrutiu I, Mouras A: **Sex organ determination and**

**differentiation in the dioecious plant Melandrium album (Silene latifolia): a cytological and histological analysis.** Sex Plant Reprod 1997, **10:**155-167.
